# STING Induces Liver Ischemia-Reperfusion Injury by Promoting Calcium-Dependent Caspase 1-GSDMD Processing in Macrophages

**DOI:** 10.1155/2022/8123157

**Published:** 2022-01-30

**Authors:** Xin-yi Wu, Ya-jun Chen, Chang-an Liu, Jun-hua Gong, Xue-song Xu

**Affiliations:** Department of Hepatobiliary Surgery, The Second Affiliated Hospital of Chongqing Medical University, Chongqing, China

## Abstract

**Objectives:**

Although a recent study reported that stimulator of interferon genes (STING) in macrophages has an important regulatory effect on liver ischemia-reperfusion injury (IRI), the underlying mechanism of STING-dependent innate immune activation in liver macrophages (Kupffer cells, KCs) remains unclear. Here, we investigated the effect of STING on liver macrophage pyroptosis and the associated regulatory mechanism of liver IRI.

**Methods:**

Clodronate liposomes were used to block liver macrophages. AAV-STING-RNAi-F4/80-EGFP, an adenoassociated virus (AAV), was transfected into the portal vein of mice in vivo, and the liver IRI model was established 14 days later. In vitro, liver macrophages were treated with STING-specific siRNA, and a hypoxia-reoxygenation (H/R) model was established. The level of STING was detected via Western blotting (WB), RT-PCR, and immunostaining. Liver tissue and blood samples were collected. Pathological changes in liver tissue were detected by hematoxylin and eosin (H&E) staining. Macrophage pyroptosis was detected by WB, confocal laser scanning microscopy (CLSM), transmission electron microscopy (TEM), and enzyme-linked immunosorbent assay (ELISA). The calcium concentration was measured by immunofluorescence and analyzed with a fluorescence microplate reader.

**Results:**

The expression of STING increased with liver IRI but decreased significantly after the clodronate liposome blockade of liver macrophages. After knockdown of STING, the activation of caspase 1-GSDMD in macrophages and liver IRI was alleviated. More interestingly, hypoxia/reoxygenation (H/R) increased the calcium concentration in liver macrophages, but the calcium concentration was decreased after STING knockdown. Furthermore, after the inhibition of calcium in H/R-induced liver macrophages by BAPTA-AM, pyroptosis was significantly reduced, but the expression of STING was not significantlydecreased.

**Conclusions:**

Knockdown of STING reduces calcium-dependent macrophage caspase 1-GSDMD-mediated liver IRI, representing a potential therapeutic approach in the clinic.

## 1. Introduction

Liver ischemia-reperfusion injury (IRI) is still a major problem affecting the survival of patients who undergo liver transplantation or partial hepatectomy [1]. It has been proposed that warm liver IRI has two distinct phases [2]. The early phase of liver injury is characterized by ATP exhaustion, mitochondrial dysfunction, and reactive oxygen species (ROS) production, which occur in hepatocytes and directly lead to hepatocyte death [3]. The second phase of liver injury is the inflammatory response, which is an important factor that contributes to liver IRI [4]; moreover, macrophages play a critical role in this process [5]. The activation of macrophages in response to pathogen-associated molecular patterns (PAMPs) or damage-associated molecular patterns (DAMPs) enhances the recruitment and activation of other innate and adaptive immune cells to amplify intrahepatic inflammation [6]. Therefore, understanding the mechanism of the innate immune response mediated by macrophages can reveal targets for the treatment of liver IRI.

Stimulator of interferon genes (STING, also known as TMEM173) is an endoplasmic reticulum- (ER-) related immune adaptor protein that can trigger a PAMP- (e.g., bacterial cyclic dinucleotide [CDN]) or DAMP-induced (e.g., DNA) inflammatory response, which has important functions in inflammation, infection, and tissue damage [7]. Recent studies have reported that the degree of liver IRI in STING-deficient mice is significantly reduced compared with that in WT mice. Moreover, aging can aggravate liver IRI by promoting STING in macrophages [8, 9]. However, little is known about the effect of STING on the death of macrophages during liver IRI or the associated mechanism.

Pyroptosis was identified as a novel form of programmed cell death that is mediated by inflammasome activation [10]. When cells undergo pyroptosis, they become swollen, organelles become deformed, and gasdermin D (GSDMD) is cleaved by activated caspase 1 to release its N-terminal domain (GSDMD-N), which in turn oligomerizes and assembles into pores in the plasma membrane, resulting in the release of a large amount of cell contents and the induction of an inflammatory response [11, 12]. Moreover, pores are formed when pyroptosis occurs, which allows the release of cytoplasmic contents, such as lactate dehydrogenase (LDH) and inflammatory cytokines; these cytokines include interleukin- (IL-) 1*β* and IL-18, which recruit immunocytes to further aggravate the inflammatory response [13]. According to a previous study [14], blocking caspase 1-GSDMD processing in innate immune cells, but not in hepatocytes, could significantly ameliorate liver injury during liver IRI and suppress the inflammatory response in innate immune cells. However, it is unclear whether STING induces macrophage pyroptosis to affect liver IRI.

Previous studies have found the ER functions as a sink for Ca^2+^ that enters cells via channels and as a store for Ca^2+^ that is released into the cytosol [15]. Excessive calcium mobilization from the ER to the cytoplasm causes activation of caspase 1, pyroptosis, and proinflammatory cytokine secretion [16]. According to a previous study [17], STING promotes ER calcium release, leading to subsequent F3 release and coagulation activation in sepsis. However, it is unclear whether STING promotes calcium release from the ER to further cause macrophage pyroptosis and tissue injury during liver IRI.

In the present study, we investigated the effect of STING in macrophages on liver IRI to further elucidate the possible mechanisms of these processes. We demonstrated that STING increases intracellular calcium in liver macrophages to promote pyroptosis, which leads to an increase in liver inflammation and tissue injury in liver IRI, and calcium promoted by STING in this process may be mainly released from the ER.

## 2. Materials and Methods

### 2.1. Animals

Male wild-type (WT) C57BL/6 mice (8–10 weeks old) were provided by the Experimental Animal Center of Chongqing Medical University (Chongqing, China). Humane care guided by the guidelines of the National Institutes of Health was provided to all animals. The protocols used in this research were evaluated and approved by the Animal Use and Ethics Committee of the 2nd Affiliated Hospital of Chongqing Medical University.

### 2.2. Liver IRI Model

A model of partial warm hepatic IRI was established as described previously [18]. In brief, the sham group had only free hepatic portal blood vessels after laparotomy without blockade of blood flow. The IRI group had the hepatic portal vein clamped, which blocked the blood supply to the left lobe and midhepatic lobe, for 90 minutes, and the blood vessels were then opened for 6 h. If a mouse died before a sample was collected, the sample was discarded. All operations were performed by the same operator, and the mice were fasted for 12 h before surgery.

Clodronate liposomes (20 mM, 200 *μ*l/per mouse; F70101C-NC, FormuMax) were administered intraperitoneally 24 h before liver IRI.AAV-Ctrl-F4/80-EGFP and AAV-STING-RNAi-F4/80-EGFP were obtained from GeneChem Company, Ltd. (Shanghai).

To confirm the specificity of F4/80-labeled AAV in liver macrophages in vivo, the fluorescence intensity in the liver and spleen (as the self-control) with or without clodronate liposome treatment (to block liver macrophages in the liver) was detected by livefluorescence imaging two weeks after transfection. As shown in Figure S1A, the fluorescence intensity in liver tissue without clodronate liposome treatment (*R*) was significantly higher than that in spleen and liver tissues treated with clodronate liposomes (*L*). However, the fluorescence intensity in spleen tissue in both groups was very low. These results suggested that F4/80-labeled AAV has a high level of liver macrophage specificity.

VX-765 (50 mg/kg) (GlpBio, USA) was administered intraperitoneally 1 h before liver IRI. In the IRI + DMSO group, a volume of 1% dimethyl sulfoxide (DMSO) equal to the volume of VX-765 was administered in the same manner.

### 2.3. Serum Biochemistry Measurements and Liver Histopathology

Serum ALT and AST levels were measured by a micromethod (Solarbio, Beijing). Some liver specimens were fixed in 4% paraformaldehyde and embedded in paraffin. Liver sections were stained with hematoxylin and eosin (H&E). The severity of liver IRI was graded using the Suzuki score. Tissues without necrosis or congestion/centrilobular ballooning were given a score of 0, whereas those presenting with severe congestion and/or > 60% lobular necrosis were given a score of 4.

### 2.4. Cell Culture and Treatment

According to the three-step approach proposed by Li [19] that includes digestion with collagenase IV (Sigma-Aldrich), gradient centrifugation, and selective adherence, Kupffer cells (KCs) were isolated from normal liver samples. The KCs were then cultured in DMEM (Gibco) supplemented with 10% FBS (Gibco), 100 U/mL penicillin G, and 100 U/mL streptomycin at 37°C in the presence of 5% CO_2_. After culturing for 24 h, the phagocytic activity of KCs was examined using an ink assay (Figure S2A). As shown in Figure S2B, the percentage of F4/80-positive cells was greater than 90%. This indicated that our method efficiently separated KCs from liver tissue while retaining normal phagocytic function.

To perform H/R in vitro, KCs were cultured at 37°C in an incubator chamber with an atmosphere of 1% O_2_, 5% CO_2_, and 94% N_2_ for 3 h, and then the KCs were returned to a normoxic incubator for 6 h.

BAPTA-AM (196419, Sigma-Aldrich, USA) was administered (10 *μ*M) for 24 h.

### 2.5. siRNA Transfection

The day before transfection, KCs were seeded and cultured in DMEM supplemented with 10% FBS, 100 U/mL penicillin G, and 100 U/mL streptomycin at 37°C in the presence of 5% CO_2_. The number of cells seeded at the initial inoculation was sufficient to reach cell confluence (70-90%) within 24 h. Then, 20 *μ*M STING siRNA (GenePharma) and Lipofectamine reagent (GenePharma) were added to equal amounts of serum-free DMEM and mixed together. The complex was added to the wells of the culture plate containing cells and culture medium and incubated for 6 h, and then the culture medium was changed to normal culture medium. After the cells were incubated in a CO_2_ incubator at 37°C for 24 h-48 h, other detection steps were performed.

### 2.6. Transmission Electron Microscopy (TEM)

Liver tissues harvested from treated mice were cut into 1 mm^3^ pieces and fixed in 2% glutaraldehyde. KCs were centrifuged (1200 rpm, 10 minutes) and then fixed with electron microscope fixative. These specimens were delivered to the Electron Microscopy Center of Chongqing Medical University and observed under TEM.

### 2.7. Immunohistochemical Staining

Paraffin sections were dewaxed using xylene and then gradient eluted with ethyl alcohol. After dewaxing and elution, the sections were digested with pancreatic enzymes at 37°C for 30 minutes. Next, the sections were boiled in citrate buffer solution for 5 minutes. The paraffin sections were blocked with goat serum at 37°C for 10 minutes, incubated with primary antibodies against STING (1 : 100, #13647, CST) and F4/80 (1 : 250, #70076, CST) at 4°C overnight, and then incubated with secondary antibodies at 37°C for 30 minutes. A visualization solution (including DAB, H_2_O_2_ and PBS) was then added to the sections for staining. Next, the sections were stained with hematoxylin at room temperature for 30 seconds. Finally, the paraffin sections were dewaxed using xylene and eluted with a gradient of ethyl alcohol. The staining of each section was observed using an inverted microscope.

### 2.8. Immunofluorescence Staining

Frozen sections of liver tissue or KCs in each group were fixed by immersion in 4% buffered formaldehyde at room temperature for 10 minutes, permeabilized with 0.2% Triton X-100 at room temperature for 30 minutes, and blocked with 1% BSA at room temperature for 1 h. PBS was used to wash the sections at room temperature for 5 minutes. Frozen sections of liver tissue or KCs were next incubated in the dark at 4°C overnight with primary antibodies against STING (1 : 300, 19851-1-AP, Proteintech), F4/80 (5 *μ*g/mL, ab6640, Abcam), and caspase 1 (1 : 300, 14F468, Novus). Next, the frozen sections or KCs were washed in the dark at room temperature for 5 minutes. The frozen sections or KCs were then incubated in the dark with a secondary antibody (1 : 500, Abcam) at room temperature for 1 h. The frozen sections or KCs were next washed in the dark at room temperature for 5 minutes. Finally, the frozen sections or KCs were incubated in the dark with DAPI (AR1176, BOSTER) at room temperature for 8 minutes. After mounting using an antifluorescence quenching agent (AR0036, BOSTER), the staining was observed using a laser scanning confocal microscope (LSCM).

### 2.9. Western Blot Analysis

#### 2.9.1. Protein Extraction and Concentration Determination

RIPA buffer tissue/cell lysate (P0013B, Beyotime) was used to extract protein from cells or tissues, and then the lysates were centrifuged at 12000 rpm for 15 minutes. The BCA Protein Assay Kit (P0010, Beyotime) was used to make a protein concentration standard to calculate the sample concentration and protein loading volume. Protein and 5× SDS loading buffer (P0286, Beyotime) were mixed at a ratio of 4 : 1 and boiled at 100°C for 10 minutes to denature the protein.

#### 2.9.2. Electrophoresis and Electrotransfer

Samples were added to a gel configured with the PAGE Gel Fast Preparation Kit (12.5%, Epizyme), then the upper gel was run at 80 V, and the lower gel was run at 100 V to the bottom. The proteins in the gel were transferred after electrophoresis to a PVDF membrane via electrotransfer at 250 mA for 60 minutes.

#### 2.9.3. Incubation with a Blocking Solution, Primary Antibody, and Secondary Antibody

The PVDF membrane was placed in a 5% BSA solution at room temperature for 1.5 h for blocking. The following primary antibodies were incubated at 4°C overnight: anti-STING (1 : 1000, #13647, CST), anti-caspase 1, anti-cleaved-caspase 1 (1 : 1000, ab179515, Abcam), anti-GSDMD, anti-GSDMD-N (1 : 1000, ab209845, Abcam), anti-tubulin (1 : 50000, 66031-1-Ig, Proteintech), and anti-*β*-actin (1 : 10000, ab6276, Abcam). At room temperature, the membrane was washed 3 times for 10 minutes in TBST. A secondary antibody was incubated for one hour at room temperature. The membrane was washed 3 times for 10 minutes with TBST at room temperature.

#### 2.9.4. Exposure and Gray Value Measurement

Signals were detected via chemiluminescence using a gel imaging system. ImageLab was used to analyze the image gray values.

### 2.10. Quantitative RT-PCR (qRT-PCR)

The RNA-Quick Purification Kit (YiShan Biotech) was used to extract RNA. An ultramicrospectrophotometer was used to measure the purity and concentration of the isolated RNA. Complementary DNA (cDNA) was synthesized using 1 *μ*g total RNA in a first-strand cDNA synthesis reaction with RT Master Mix for qPCR (MCE). RT-PCR was performed using a CFX 96 qPCR system (Bio-Rad). The SYBR RT-PCR Kit (MCE) was used for qRT-PCR analysis. All samples were normalized according to the *β*-actin expression. The results were statistically analyzed using the 2^-△△CT^ method.

The primers used for RT-PCR analysis were as follows: STING forward: CCGAAGACTGTACATCCTCTTT, STING reverse: AGCATATCTCGGAATCGAATGT. *β*-Actin forward: CTACCTCATGAAGATCCTGACC, *β*-actin reverse: CACAGCTTCTCTTTGATGTCAC.

### 2.11. Caspase 1 Activity

Caspase 1 activity in liver tissues and KCs was detected. The activity was measured with the Caspase 1 Assay Kit (BC3810, Solarbio) according to the manufacturer's instructions.

### 2.12. Measurement of Intracellular Calcium Levels

According to the manufacturer's instructions, KCs were treated with Fluo-4 AM (2 *μ*M) at 37°C for 30 minutes in the dark. A fluorescence microplate reader was used to detect intracellular calcium signals (the excitation wavelength was 480 nm, and the emission wavelength was 520 nm). After incubation with Hoechst stain for 30 minutes at 37°C, the fluorescence intensity of cells was observed with an inverted fluorescence microscope.

### 2.13. Enzyme-Linked Immunosorbent Assay (ELISA)

ELISA kits were used to detect IL-1*β* and IL-18 (Jiubang Biotechnology) in mouse serum or KC supernatant according to the manufacturer's protocols.

### 2.14. LDH Assays

Liver tissues or KCs were treated as described above, and cytotoxicity was quantitated by measuring LDH using the LDH Activity Assay Kit (BC0685, Solarbio) according to the manufacturer's instructions.

### 2.15. Statistical Analysis

All results were analyzed using SPSS 18.0 software (SPSS Inc., Chicago, USA). Normally distributed data are shown as the mean ± SD. Differences between groups were evaluated using a *t*-test. The Shapiro-Wilk test was used to test for normality. Data exhibiting a *P* value >0.05 were regarded as conforming to a normal distribution. Nonnormally distributed data are shown as the median, and differences were evaluated using the rank-sum test. Differences with *P* values <0.05 were regarded as statistically significant.

## 3. Results

### 3.1. STING Is Increased in Macrophages during Liver IRI

**Figure 1:**
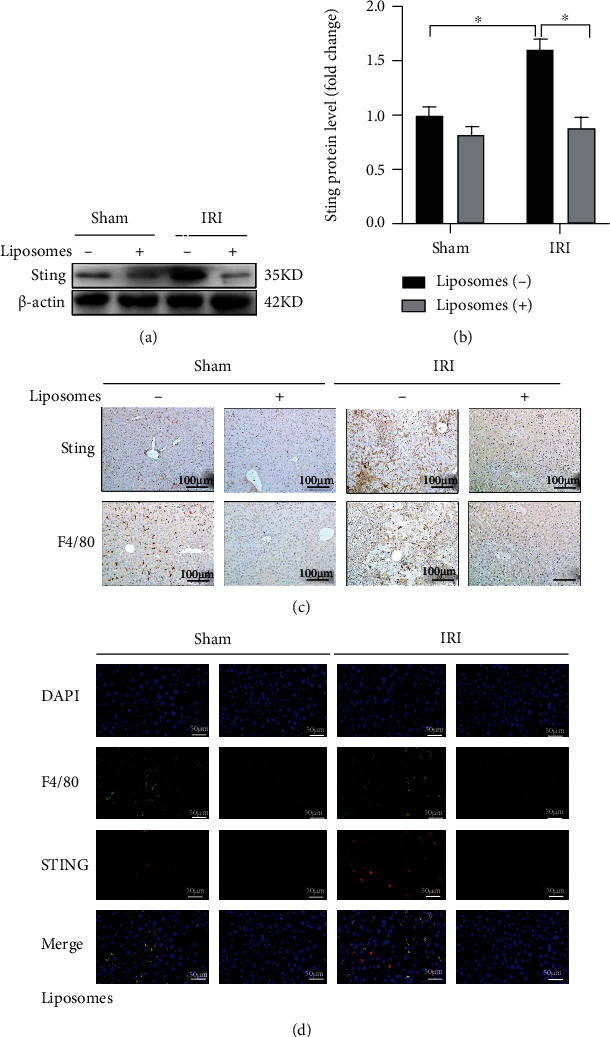
The expression of STING in macrophages was induced in liver IRI.C57/B6 mice were subjected to liver IRI. (a) STING expression in the sham group and the IRI group in the presence or absence of liposomes (20 mM, 200 *μ*l/per mouse) was measured by Western blotting. (b) Relative expression of STING in each group. (c) STING expression in each group was measured by immunohistochemistry (scale bar, 50 *μ*m). (d) The colocalization of F4/80 and STING in KCs was measured by immunofluorescence in each group (scale bar, 100 *μ*m). All data are shown as the mean ± SD (*n* = 6 mice per group). ^∗∗∗^*P* < 0.001, ^∗∗^*P* < 0.01, and ^∗^*P* < 0.05.

STING is a universal receptor that recognizes released DNA and triggers innate immune activation, which has important functions in infection, inflammation and cancer [20]. To investigate whether STING is involved in liver IRI, we first examined the expression of STING in liver tissues in a liver IRI mouse model. As shown in Figures 1(a) and 1(b), compared to that in the sham group, the expression of liver STING in the IRI group was significantly increased. Additionally, both immunohistochemistry (Figure 1(c)) and immunofluorescence (Figure 1(d)) results showed that the STING-positive areas in the IRI group were significantly larger than those in the sham group.

STING signaling regulates macrophage proinflammatory activation and liver IRI [8, 21]. Thus, we blocked macrophages by injecting the macrophage inhibitor liposome-encapsulated clodronate and then observed the expression of STING. Notably, the results generated by Western blotting (WB) (Figures 1(a) and 1(b)), immunohistochemistry (Figure 1(c)), and immunofluorescence (Figure 1(d)) showed that the expression of STING was significantly reduced in the IRI group after liposomes were added. These results preliminarily suggest that STING in activated macrophages is involved in the process of liver IRI.

### 3.2. H/R Induces the Activation of STING in Liver Macrophages

**Figure 2:**
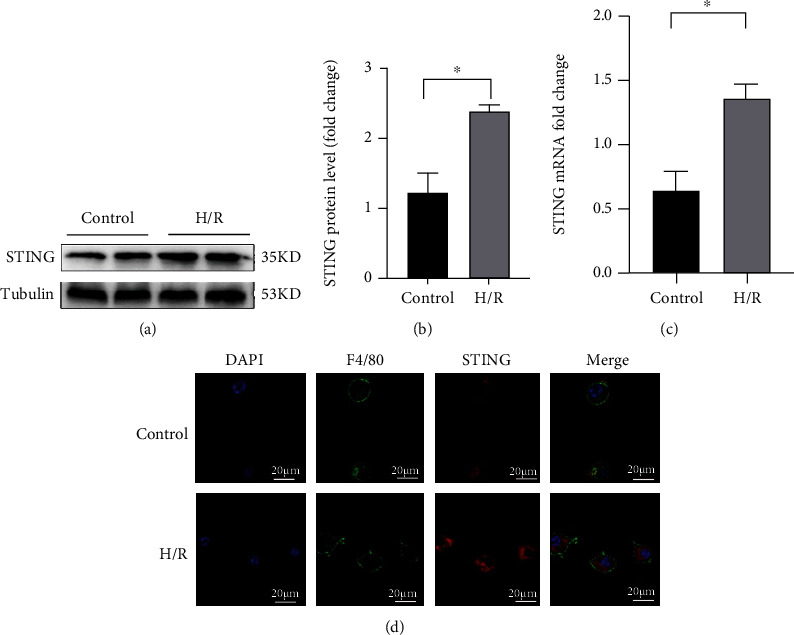
The expression of STING in liver macrophages was induced by hypoxia-reoxygenation (H/R) treatment. KCs were isolated from the liver and treated with H/R. (a) STING expression in the control group and the H/R group was measured by Western blotting. (b) Relative expression of STING in each group. (c) The mRNA levels of STING were measured by quantitative real-time PCR. (d) The colocalization of F4/80 and STING in KCs was measured by confocal laser scanning microscopy (CLSM) in each group (scale bar, 20 *μ*m). All data are shown as the mean ± SD (*n* = 6 mice per group). ^∗∗∗^*P* < 0.001, ^∗∗^*P* < 0.01, and ^∗^*P* < 0.05.

We further investigated the cellular origin of STING in the liver. Macrophages were isolated from liver tissues and treated with H/R in vitro. The expression of STING in macrophages in the H/R group was significantly higher than that in macrophages in the control group (Figures 2(a) and 2(b)). The mRNA levels of STING in liver macrophages in the H/R group were also significantly higher than those in the control group (Figure 2(c)). Furthermore, confocal laser scanning microscopy (CLSM) (Figure 2(d)) results showed that the STING-positive areas (labeled in red) in the H/R group were significantly more intense than those in the control group when liver macrophages in each group were labeled with F4/80 (green). Our results reveal that STING is expressed primarily in liver macrophages and may be important in the context of liver IRI.

### 3.3. Knockdown of STING in Macrophages Alleviates Liver IRI

To further dissect the effects of STING in macrophages during liver IRI, we used an EGFP-F4/80-labeled AAV to knock down STING in macrophages in vivo to observe whether knockdown of STING exerts a protective effect against liver IRI.

**Figure 3:**
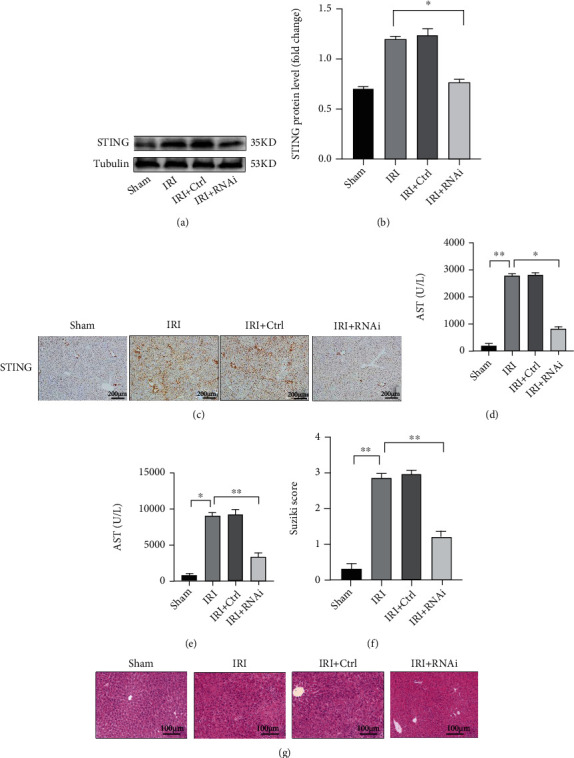
Knockdown of STING in macrophages alleviated liver IRI. Mice were pretreated (i.v.) with AAV-STING-RNAi-F4/80-EGFP (1.5 × 10^11^ vg) 14 days before liver IRI modeling. A volume of nonspecific AAV (AAV-Ctrl-F4/80-EGFP) equal to the treatment volume was administered in the same manner.(a) KCs were isolated from the liver in each group, and then the expression of STING in KCs in each group was measured by Western blotting. (b) Relative STING expression of KCs in each group. (c) STING expression in each group was measured by immunohistochemistry (scale bar, 200 *μ*m). (d, e) Serum levels of ALT and AST were measured. (f, g) H&E-stained sections of livers; average Suzuki scores were based on H&E-stained liver sections from different groups of mice (scale bar, 100 *μ*m). All data are shown as the mean ± SD (*n* = 6 mice per group). ^∗∗∗^*P* < 0.001, ^∗∗^*P* < 0.01, and ^∗^*P* < 0.05.

As shown in Figures 3(a) and 3(b), liver macrophages were isolated from the livers in each group, and the expression of STING in liver macrophages in the IRI + Ctrl group was not significantly altered compared to that in the IRI group; however, the expression of STING in liver macrophages in the IRI + RNAi group was significantly lower than that in those of the IRI group. This result indicated that AAV-RNAi effectively knocked down STING in macrophages during liver IRI. Moreover, immunohistochemistry (Figure 3(c)) also showed that the expression of STING in IRI model mice was significantly reduced after the injection of AAV-RNAi. Compared to those in the sham group, the serum ALT and AST levels in the IRI group were significantly elevated. There were no significant differences in these values between the IRI + Ctrl group and the IRI group. However, the ALT and AST levels in the IRI + RNAi group were significantly lower than those in the IRI group (Figures 3(d) and 3(e)). Furthermore, H&E staining and Suzuki scores indicated that the damage to liver tissue in the IRI group was significantly more severe than that in the sham group, while the liver tissue structure of the IRI + RNAi group was better preserved, and the Suzuki score in the IRI + RNAi group was lower than that in the IRI group (Figures 3(f) and 3(g)). In general, we observed that knockdown of STING in macrophages alleviated the degree of liver IRI and improved the levels of liver function markers, all of which were beneficial for alleviating the damage caused by liver IRI.

### 3.4. STING Aggravates Inflammation by Promoting Caspase 1-Dependent GSDMD Activation in H/R-Induced Liver Macrophages

Pyroptosis, a proinflammatory form of programmed cell death, in innate immune cells aggravates liver IRI, implying that liver IRI-related damage can be mitigated by blocking pyroptosis in macrophages, which may become a potential therapeutic approach in the clinic [22]. Liver macrophages act as innate immune cells and are an important source of inflammatory factors in the liver [23]. Therefore, to further explore the effect of STING on liver macrophages, we tested whether STING affects pyroptosis in liver macrophages and the production of inflammatory factors.

**Figure 4:**
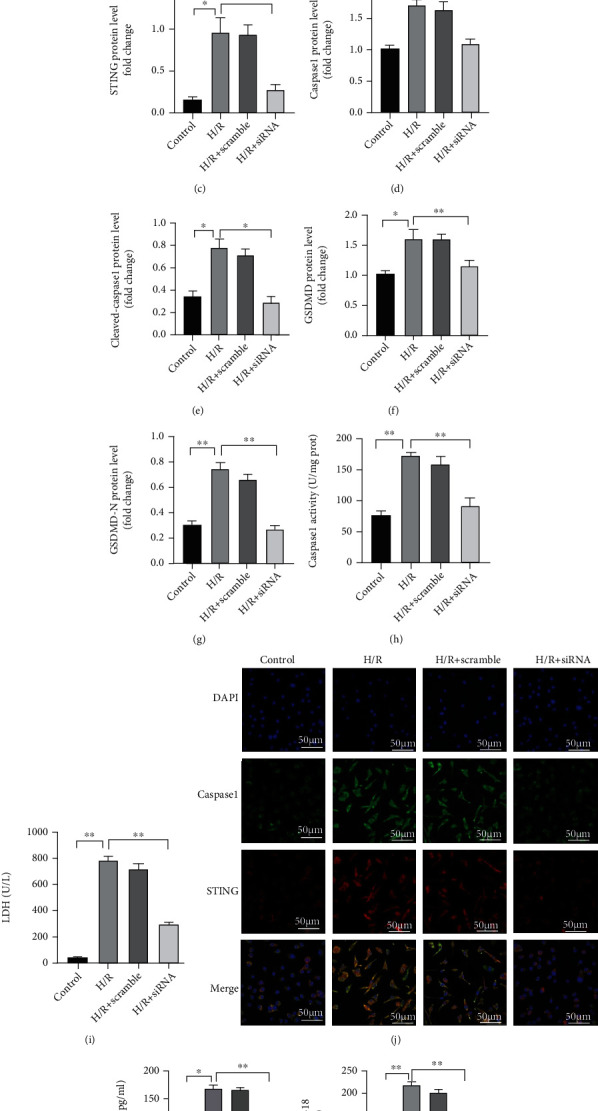
Knockdown of STING in liver macrophages reduces caspase 1-GSDMD expression and H/R-induced inflammation. KCs were isolated from the liver and pretreated with STING-specific siRNA (20 *μ*M) or nonspecific siRNA (scramble) before H/R modeling. (a) The levels of STING, caspase 1, cleaved-caspase 1, GSDMD, and GSDMD-N in each group were measured by Western blotting. (b) The mRNA levels of STING were measured by quantitative RT-PCR. (c)–(g) Relative expression of STING, caspase 1, cleaved-caspase 1, GSDMD, and GSDMD-N in each group. (h) Caspase 1 activity was measured with a caspase 1 assay kit. (i) Supernatant LDH levels were measured. (j) The colocalization of caspase 1 and STING in KCs was measured by confocal laser scanning microscopy in each group (scale bar, 50 *μ*m). (k, l) The levels of cytokines (IL-1*β* and IL-18) in the cell culture supernatant were measured by ELISA. All data are shown as the mean ± SD, (*n* = 6). ^∗∗∗^*P* < 0.001, ^∗∗^*P* < 0.01, and ^∗^*P* < 0.05.

We isolated macrophages from the liver and transfected them with STING-specific siRNA. Then, liver macrophages were treated with H/R in vitro. The mRNA levels of STING in liver macrophages in the H/R+ siRNA group were significantly lower than those in the H/R group (Figure 4(b)). Compared with that in the control group, the expression of STING, procaspase 1, cleaved-caspase 1, GSDMD, and GSDMD-N in liver macrophages in the H/R group was increased, but there were no significant changes compared with that in the H/R + scramble group. The expression levels of STING, caspase 1, cleaved-caspase 1, GSDMD, and GSDMD-N in liver macrophages in the H/R + siRNA group were significantly lower than those in the H/R group (Figures 4(a) and 4(c)–4(g)). Furthermore, decreased caspase 1 activity was also observed after STING-specific siRNA treatment (Figure 4(h)). Liver macrophages were treated with H/R, and STING (red), and caspase 1 (green) were labeled. The fluorescence intensities of STING and caspase 1 in liver macrophages in the H/R group were significantly higher than those in the control group. However, after STING-specific siRNA treatment knocked down the expression of STING in liver macrophages, the fluorescence intensities of STING (red) and caspase 1 (green) were significantly reduced (Figure 4(j)).

Functionally, the proinflammatory effect of H/R-treated liver macrophages was enhanced, and the levels of IL-1*β*, IL-18, and LDH in the supernatant were significantly increased; however, after STING-specific siRNA treatment knocked down STING in H/R-treated liver macrophages, the levels of IL-1*β* and IL-18 in the supernatant were significantly reduced (Figures 4(i), 4(k), and 4(l)). These results show that STING in liver macrophages promotes the secretion of inflammatory cytokines, and that this process is related to caspase 1-GSDMD.

### 3.5. STING-Mediated Caspase 1-GSDMD Processing in Macrophages Promotes Liver IRI

**Figure 5:**
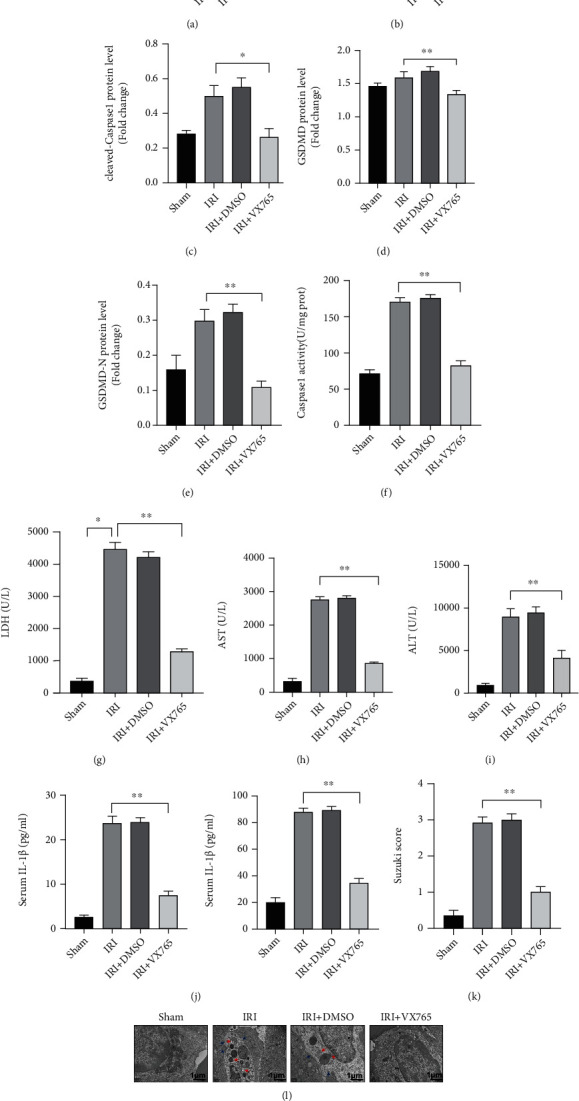
Inhibition of caspase 1 attenuates liver IRI mediated by GSDMD in macrophages. VX-765 (50 mg/kg) was administered intraperitoneally 1 h before liver ischemia. C57/B6 mice were subjected to liver IRI modeling, and KCs were isolated from the liver in each group. (a) The levels of caspase 1, cleaved-caspase 1, GSDMD, and GSDMD-N in each group were measured by Western blotting. (b)–(e) Relative expression of caspase 1, cleaved-caspase 1, GSDMD, and GSDMD-N in each group. (f) Caspase 1 activity was measured with a caspase 1 assay kit. (g)–(i) Serum levels of ALT, AST, and LDH were measured. (j) The levels of cytokines (IL-1*β* and IL-18) in the cell culture supernatant were measured by ELISA. (k, l) H&E-stained sections of livers; average Suzuki scores were based on H&E-stained liver sections from different groups of mice (scale bar, 100 *μ*m). (m) Transmission electron microscopy (TEM) was used to observe the ultrastructural changes in KCs (original magnification, ×20000). The red arrow indicates the incomplete structure of an organelle, and the blue arrow indicates discontinuity in the cell membrane. All data are shown as the mean ± SD (*n* = 6 mice per group). ^∗∗∗^*P* < 0.001, ^∗∗^*P* < 0.01, and ^∗^*P* < 0.05.

Previous studies have shown that VX-765 (caspase 1 inhibitor) is a small-molecule inhibitor that inhibits both the expression and activity of caspase 1 [24]. More importantly, GSDMD processing may not occur in hepatocytes during liver IRI, and VX-765 has no protective effects on hepatocytes during H/R treatment [14]. Therefore, we further explored whether STING promotes liver IRI by inducing macrophage pyroptosis. VX-765 (50 mg/kg) was administered intraperitoneally 1 h before liver IRI modeling, and then KCs were isolated from the liver in each group. As shown in Figures 5(a)–5(e), VX-765 eliminated the increased expression of caspase 1, cleaved-caspase 1, GSDMD, and GSDMD-N in macrophages after liver IRI. Moreover, after mice were treated with VX-765, the activity of caspase 1 in macrophages was significantly reduced (Figure 5(f)). The LDH, ALT, and AST levels in the IRI group were significantly higher than those in the sham group; however, after treatment with VX-765, the LDH, ALT, and AST levels were significantly decreased (Figures 5(g)–5(i)). As shown in Figure 5(j), IRI further promoted the secretion of IL-1*β* and IL-18 in the liver, while VX-765 significantly inhibited the secretion and release of these inflammatory factors. It is worth noting that the degree of liver tissue damage in the IRI group was more serious than that in the sham group, but this liver tissue damage was significantly reduced after VX-765 treatment (Figures 5(k) and 5(l)). Furthermore, the ultrastructures of liver tissues were evaluated by TEM. Compared with those in the sham group, the liver macrophages in the IRI group were swollen, the cell membrane was ruptured, pores were present, and the structure of the mitochondria and lysosomes was incomplete. However, after treatment with VX-765, liver macrophages were not significantly swollen, the cell membrane was continuous, and the structure of each organelle was relatively complete (Figure 5(m)). These results suggest that STING probably affects liver IRI by regulating macrophage pyroptosis.

### 3.6. STING Increases Intracellular Calcium in H/R-Induced Liver Macrophages

**Figure 6:**
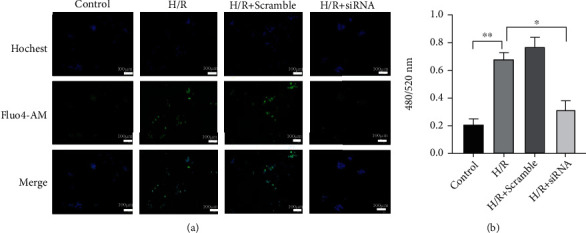
Knockdown of STING reduces the intracellular calcium concentration in H/R-induced liver macrophages. KCs were isolated from the liver and pretreated with STING-specific siRNA (20 *μ*M) or nonspecific siRNA (Scramble) before H/R modeling. (a) The concentration of calcium was measured by immunofluorescence, where intracellular calcium was labeled with Fluo-4 AM (green), and the nucleus was labeled with Hoechst stain (scale bar, 100 *μ*m). (b) The relative concentration of calcium in each group was measured with a fluorescence microplate reader. All data are shown as the mean ± SD (*n* = 6). ^∗∗∗^*P* < 0.001, ^∗∗^*P* < 0.01, and ^∗^*P* < 0.05.

Calcium signaling is a universal and versatile mechanism involved in a wide range of fundamental cellular events. Increased cytosolic calcium influx from the ER is an endogenous signal that drives cell death and immune responses [25]. Given that STING is an ER-associated membrane protein [7], we determined whether the changes in intracellular calcium concentration were related to STING. As shown in Figures 6(a) and 6(b), the calcium concentration in liver macrophages treated with H/R was significantly increased, but after STING was knocked down in liver macrophages, the calcium concentration was significantly decreased. These findings show that H/R-induced STING increases intracellular calcium in liver macrophages.

### 3.7. H/R-Induced STING Increases Intracellular Calcium to Promote Caspase 1-GSDMD Processing in Liver Macrophages

**Figure 7:**
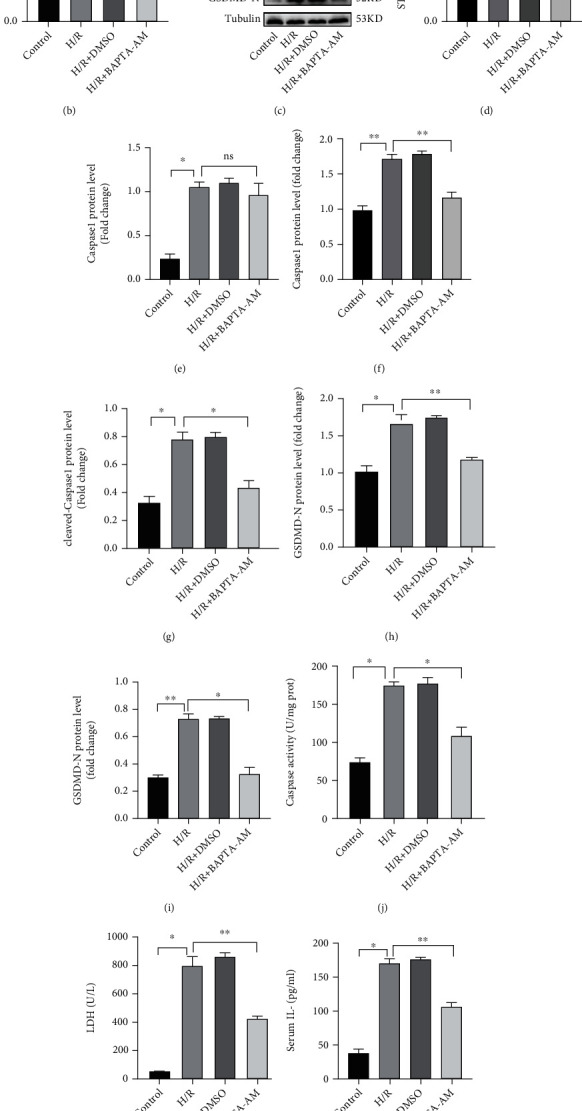
H/R-induced STING increases intracellular calcium to promote caspase 1-GSDMD processing in liver macrophages. KCs were isolated from the liver and treated with H/R in the absence or presence of BAPTA-AM (10 *μ*M) for 24 h.(a) The concentration of calcium was measured by immunofluorescence, where intracellular calcium was labeled with Fluo-4 AM (green), and the nucleus was labeled with Hoechst stain (scale bar, 100 *μ*m). (b) The relative concentration of calcium in each group was measured with a fluorescence microplate reader. (c) The levels of STING, caspase 1, cleaved-caspase 1, GSDMD, and GSDMD-N in each group were measured by Western blotting. (d) The mRNA levels of STING were measured by quantitative RT-PCR. (e)–(i) Relative expression of STING, caspase 1, cleaved-caspase 1, GSDMD, and GSDMD-N in each group. (j) Caspase 1 activity was measured with a caspase 1 assay kit. (k) Supernatant LDH levels were measured. (l, m) The levels of cytokines (IL-1*β* and IL-18) in the cell culture supernatant were measured by ELISA. (n) Transmission electron microscopy (TEM) was used to observe the ultrastructural changes in KCs (original magnification, ×20000). The red arrow indicates the incomplete structure of an organelle, and the blue arrow indicates discontinuity in the cell membrane. All data are shown as the mean ± SD (*n* = 6). ^∗∗∗^*P* < 0.001, ^∗∗^*P* < 0.01, and ^∗^*P* < 0.05.

To investigate whether H/R-induced STING promotes caspase 1-GSDMD processing by increasing intracellular calcium in liver macrophages, we used BAPTA-AM (an administration of a calcium chelator) to inhibit intracellular calcium signaling in H/R-induced liver macrophages. As shown in Figures 7(a) and 7(b), the calcium concentration in the BAPTA-AM group was significantly lower than that in the HR group. Moreover, compared with that in the H/R group, the expression of caspase 1, cleaved-caspase 1, GSDMD, and GSDMD-N in the H/R + BAPTA-AM group was significantly decreased (Figures 7(c) and 7(f)–7(i)). The activity of caspase 1 in the H/R + BAPTA-AM group was lower than that in the H/R group (Figure 7(j)). Functionally, the levels of IL-1*β*, IL-18, and LDH in the supernatant were significantly decreased in the H/R+ BAPTA-AM group (Figures 7(k)–7(m)). The ultrastructures of liver macrophages were observed by TEM, and compared with those in the H/R group, liver macrophages in the H/R + BAPTA-AM group were less swollen, and the incidence of cell membrane pores was reduced and the organelle structure was more complete (Figure 7(n)). However, compared with that in the H/R group, the expression of STING in the H/R + BAPTA-AM group was slightly but not significantly reduced (Figure 7(c) and 7(e)). After intracellular calcium was inhibited by BAPTA-AM, the mRNA of STING was also not significantly decreased (Figure 7(d)).

In addition, the ER functions as a sink for calcium that enters cells via channels and as a store for calcium that is released into the cytosol [15]. Therefore, to explore whether the increased calcium during H/R is related to the ER, we treated liver macrophages with H/R in vitro and then the inhibition of inositol 1,4,5-trisphosphate receptor type 1 (ITPR1, also known as IP3R, the primary calcium release channel of the ER) [26] by siRNA or by a pharmacological inhibitor (2-APB) blocked the calcium release channel of the ER. As shown in Figure S3A and B, compared with the control group, calcium increased in the H/R group, but after blocking the ER calcium channel, calcium was significantly reduced. These results suggest that in the H/R model, the increased calcium mainly comes from the ER.

**Figure 8:**
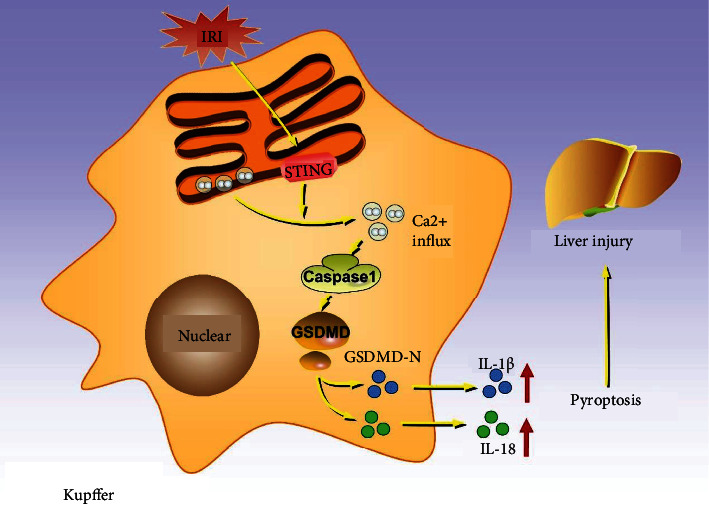
Diagram representing the putative mechanism by which STING induces liver IRI by promoting calcium-dependent pyroptosis of macrophages. During liver IRI, STING in liver macrophages increased intracellular calcium to promote caspase 1-GSDMD processing, and then IL-1*β* and IL-18 were secreted and released, which ultimately aggravated liver injury.

In conclusion, our experimental results suggest that H/R activates STING in liver macrophages. Then, H/R-induced STING increases intracellular calcium to promote pyroptosis of liver macrophages, and in this process, the increased calcium may be released from the ER (Figure 8).

## 4. Discussion

Innate immunity is the first line of defense against infection, but its excessive activation can result in tissue injury and host lethality [27, 28].STING has previously been shown to regulate inflammation and infection in health and disease [20, 29]. At present, only a few articles have reported the role of STING in liver IRI [30, 31], and the underlying mechanism remains to be determined. In this study, we found that the activation of STING in liver IRI mainly depends on liver macrophages, and that STING promotes the processing of caspase 1-GSDMD in macrophages to aggravate liver IRI. In this process, STING increases intracellular calcium to promote caspase 1-GSDMD processing, which may be related to ER stress. These findings provide a new regulatory mechanism for macrophage innate immune activation during liver IRI.

Recent studies have shown that in IRI-stressed hepatocytes, mitochondrial DNA is elevated and released, but STING signaling in hepatocytes is only slightly upregulated. In contrast, liver-derived macrophages significantly increase the STING protein level under IRI induction [8]. Moreover, hepatocytes do not express STING under normoxic conditions or after anoxia/reoxygenation (A/R) [9]. We also demonstrated that STING promotes liver IRI and mainly depends on activated liver macrophages. The differences were that we used liposome-encapsulated clodronate to block the function of liver macrophages and an EGFP-F4/80-labeled AAV to directly knockdown STING in liver macrophages to observe the expression of STING and liver injury.

Early studies revealed that STING is essential in the immune responses to bacterial and viral invasion. STING signaling can also be activated by self-DNA in necrotic cells, which subsequently initiates autoinflammatory diseases. Specifically, cytosolic DNA species can bind to cyclic GMP–AMP synthase (cGAS), leading to the production of a type of cyclic dinucleotide (CDN) [32, 33]. We found that under H/R stimulation, the expression of STING in macrophages increased, which indicated that H/R can induce the activation of STING in liver macrophages.

IRI occurs primarily following the return of blood flow and oxygen to the hypoxic liver during reperfusion. The early phase of liver injury is characterized by ATP exhaustion, mitochondrial dysfunction and reactive oxygen species production, which occur in hepatocytes and directly lead to hepatocyte death [3]. The second phase of I/R injury is characterized by the activation of innate immune cells, including Kupffer cells and neutrophils, by released DAMPs from injured or dead hepatocytes, resulting in elevated proinflammatory cytokine production and aggravated liver damage [34]. In addition, H/R-induced KCs secrete ROS or other hepatocytotoxic products [35]. Furthermore, H/R-induced PP hepatocellular injury was mediated by KC-derived O2-, while endogenously generated NO by eNOS restrained the development of the hypoxic injury response [36]. However, the mechanism of the interaction between hepatocytes and macrophages in H/R is still not fully elucidated. Therefore, this process requires further research.

In this process, massive amounts of ROS are produced and contribute to the pathogenesis of IRI. Excessive ROS production compromises intracellular components such as phospholipids, proteins, and DNA [37]. mROS generation mainly increases in the stage of reperfusion after ischemia. The loss of Mfn2 suppresses the generation of both ROS and the transcription factors associated with oxidative metabolism in endothelial cells [38]. Furthermore, cardioprotective properties are closely associated with the decreased transfer of Ca2+ from the ER into mitochondria, as Mfn2 deletion disrupts the association between these organelles [39]. Excessive mitochondrial fission induces cytosolic calcium overload and thus promotes cardiomyocyte death and myocardial contractile dysfunction [40]. During oxidative stress, endogenous nucleic acids, such as mitochondrial DNA and nuclear DNA, are released into the cytosol and circulation [41]. At the subcellular level, PGAM5 deficiency increased mitochondrial DNA copy number and transcript levels, normalized mitochondrial respiration, repressed mitochondrial ROS production, and prevented abnormal mPTP opening upon I/R [42]. Moreover, transcription factor A, mitochondria (TFAM), and HMGB1 can both orient DNA for more efficient DNA binding [43].

STING was originally identified as a cytosolic nucleic acid sensor that activates the innate immune response by inducing a type I IFN response [44, 45]. STING can activate TBK1 and IRF3, resulting in enhanced expression of type I IFNs and proinflammatory cytokines [46–48] and apoptosis [49, 50]. Classical STING-mediated type I IFN production requires STING to be transported to ERGIC, which can be regulated by certain proteins (such as ZDHHC1 and STIM1) in a calcium-independent manner [51–54]. Luo et al. [55] found that liver tissues from patients with nonalcoholic fatty liver disease and mice with high-fat diet- (HFD-) induced steatosis expressed higher levels of STING, while STING inhibition in macrophages decreased inflammation and the severity of liver fibrosis. We discovered an alternative STING pathway that leads to the activation of innate immunity in macrophages and contributes to liver IRI promotion.

Pyroptosis is a form of lytic cell death programmed by inflammatory caspases and plays a fundamental role in the antimicrobial response [56]. As recent studies have drawn few conclusions about the relationship between STING signaling and pyroptosis [57–59], it is still entirely unclear how macrophage STING triggers macrophage pyroptosis in liver IRI. Interestingly, we established an in vivo mouse model of liver IRI and an in vitro model of liver macrophages treated with H/R and found that STING triggers caspase 1-dependent GSDMD activation in macrophages to aggravate liver IRI. Pyroptosis is mediated by caspase 1 and canonical inflammasomes or caspase 11 and noncanonical inflammasomes. Recent studies showed that the protein levels and activity of caspase 1, but not those of caspase 11, were remarkably enhanced during hepatic IRI, suggesting activation of the canonical pyroptotic signaling pathway [14, 60]. HMGB1 is a ubiquitously expressed DNA-binding protein and a key endogenous DAMP [61]. HMGB1 may bind to a variety of TLRs, including TLR2, TLR4, and TLR9, to initiate an array of inflammatory responses [62]. HMGB1 release was also reported to occur in ischemia-stressed cells [63]. HMGB1 activates caspase 1 through TLR4, and activated caspase 1 cleaves pro-IL-1*β* and pro-IL-18 into mature IL-1*β* and IL-18, respectively, thereby enhancing the inflammatory response [64]. In addition, silencing TLR4 attenuates LPS-induced liver injury through inhibition of inflammation and apoptosis via the TLR4/MyD88/NF-*κ*B signaling pathway [65].

Our study highlights that STING controls calcium influxes to mediate pyroptosis in H/R-induced liver macrophages. Calcium is an intracellular second messenger, and the ER is the largest calcium storage organelle in eukaryotic cells [66]. Given that STING is an ER-related membrane protein [67], we knocked down STING in H/R-induced liver macrophages and found that the calcium signal intensity in macrophages was decreased. Then, we inhibited intracellular calcium signaling in H/R-induced liver macrophages and found that pyroptosis was significantly reduced; however, the expression of STING was slightly but not significantly reduced. We demonstrated that H/R-induced STING increases intracellular calcium to promote caspase 1-GSDMD processing in liver macrophages, and in this process, the increased calcium may be released from the ER. ER stress facilitates the UPR and contributes to the etiology of steatosis, nonalcoholic steatohepatitis, and, ultimately, hepatocarcinoma [68]. ER stress-inducing agents, in certain cell lines, lead to sustained Ca2+ release from the ER [69]. Excessive and/or sustained Ca2+ mobilization from the ER to the mitochondria results in mitochondrial Ca2+ overload, triggering leucine-rich repeat containing protein 3 (NLRP3) inflammasome activation [70]. Microvascular protective effects of sarcoplasmic/endoplasmic reticulum Ca2 + -ATPase (SERCA) on the reperfused heart. SERCA overexpression attenuates lumen stenosis, inhibits microthrombus formation, reduces the inflammatory response, and improves endothelium-dependent vascular relaxation [71]. Spontaneously elevated TNF levels were previously observed in aged mice and found to be critical for increased NLRP3 expression and caspase 1 activity in liver tissues [72]. Bone marrow-derived macrophages were shown to have higher levels of NLRP3 inflammasome activation and caspase 1-dependent IL-1*β* and IL-18 production [73]. Therefore, STING promotes calcium influx to induce macrophage caspase 1-GSDMD activation and may be involved in the NLRP3 inflammasome. In addition, the activation of GSDMD-N triggers pyroptosis and requires lipid peroxidation-mediated PLCG1 activation and subsequent calcium influx [74]. As a negative feedback mechanism resulting from calcium influx, the activation of endosomal sorting complexes required for transport (ESCRT)-dependent membrane repair could limit oxidative injury to the plasma membrane and promote cell survival [75]. Moreover, GSDMD-mediated potassium efflux can limit STING-mediated type I IFN production in response to bacterial dsDNA [58].

## 5. Conclusions

In summary, targeting STING to inhibit liver macrophage pyroptosis and excessive proinflammatory activation will be a feasible treatment or preventative method for patients with liver IRI.

## Data Availability

All data included in this study are available upon request by contacting the corresponding author.

## Supplementary Materials

Supplementary Materials Figure S1: the cell-specific identification of AAV-transfected cells was detected byliving fluorescence imaging. L: KCs were blocked by clodronate liposome treatment. R: KCs were preserved without clodronate liposome treatment. Due to the injection of AAV through the portal vein system, spleen tissue was selected as the main self-control. Figure S2: the phagocytic activity and purity of KCs. (a) The phagocytic activity of KCs was examined using an ink assay. (b) The percentage of F4/80-positive cells was detected by flow cytometry. Figure S3: the increased calcium in H/R-induced liver macrophages is mainly released from ER KCs that were treated with H/R and blocked by IP3 siRNA (20 *μ*M) or 2-APB (100 *μ*M, 24 h). (a) The concentration of calcium was measured by immunofluorescence, where intracellular calcium was labeled with Fluo-4 AM (green), and the nucleus was labeled with Hoechst stain (scale bar, 100 *μ*m). (b) The relative concentration of calcium in each group was measured with a fluorescence microplate reader. All data are shown as the mean ± SD (*n* = 6). ^∗∗∗^*P* < 0.001, ^∗∗^*P* < 0.01, and ^∗^*P* < 0.05.
