# Supplementary material for: STING Induces Liver Ischemia-Reperfusion Injury by Promoting Calcium-Dependent Caspase 1-GSDMD Processing in Macrophages
Source: Oxid Med Cell Longev. 2022 Jan 30;2022:8123157. doi: 10.1155/2022/8123157 (PMC8906939; doi:10.1155/2022/8123157)
Supplement: Supplementary Materials — Figure S1: the cell-specific identification of AAV-transfected cells was detected byliving fluorescence imaging. L: KCs were blocked by clodronate liposome treatment. R: KCs were preserved without clodronate liposome treatment. Due to the injection of AAV through the portal vein system, spleen tissue was selected as the main self-control. Figure S2: the phagocytic activity and purity of KCs. (a) The phagocytic activity of KCs was examined using an ink assay. (b) The percentage of F4/80-positive cells was detected by flow cytometry. Figure S3: the increased calcium in H/R-induced liver macrophages is mainly released from ER KCs that were treated with H/R and blocked by IP3 siRNA (20 μM) or 2-APB (100 μM, 24 h). (a) The concentration of calcium was measured by immunofluorescence, where intracellular calcium was labeled with Fluo-4 AM (green), and the nucleus was labeled with Hoechst stain (scale bar, 100 μm). (b) The relative concentration of calcium in each group was measured with a fluorescence microplate reader. All data are shown as the mean ± SD (n = 6). ∗∗∗P < 0.001, ∗∗P < 0.01, and ∗P < 0.05. [file 8123157.f1.pdf]

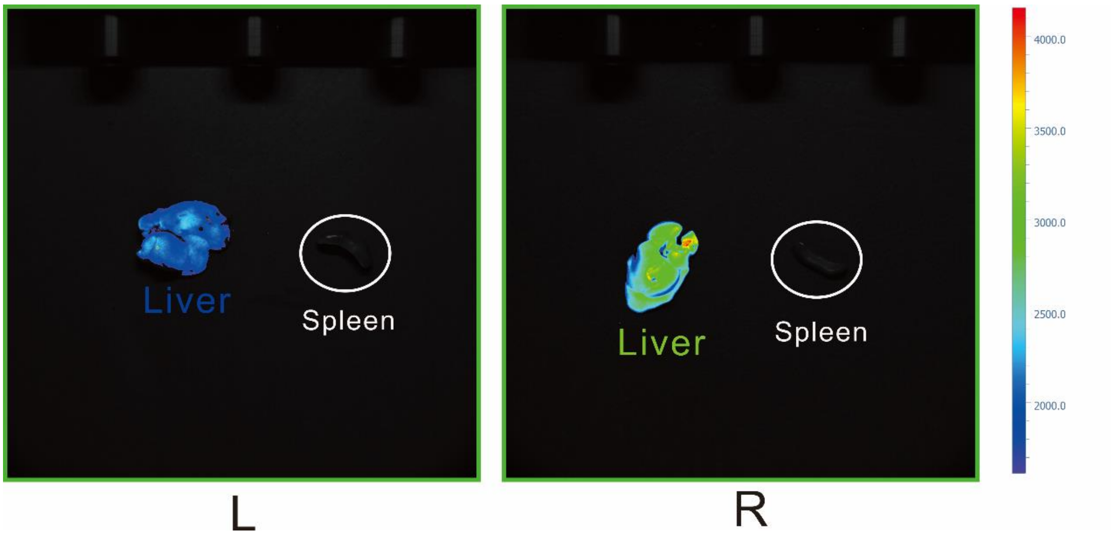

FIGURE S1. The cell-specific identification of AAV-transfected cells was detected by living fluorescence imaging. L: KCs were blocked by clodronate liposome treatment. R: KCs were preserved without clodronate liposome treatment. Due to the injection of AAV through the portal vein system, spleen tissue was selected as the main self-control.

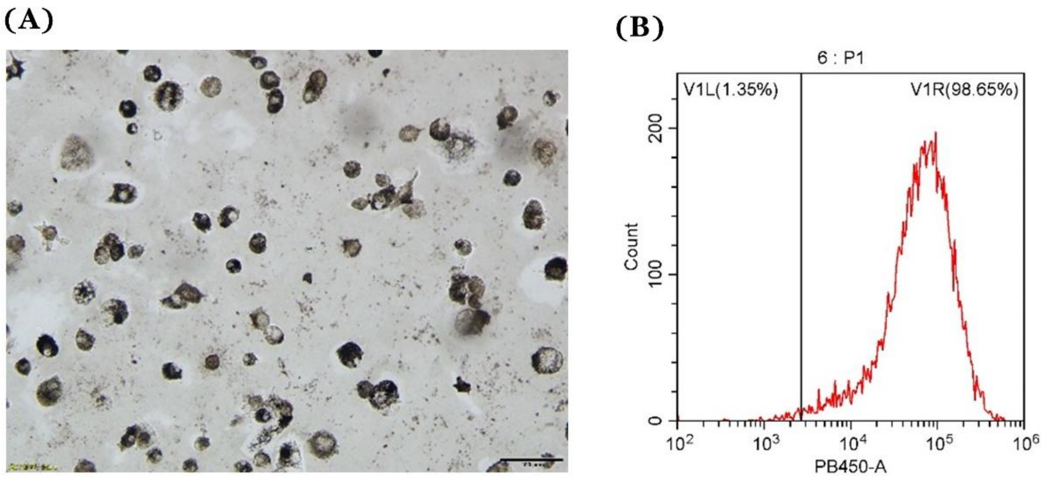

FIGURE S2 The phagocytic activity and purity of KCs. (A) The phagocytic activity of KCs was examined using an ink assay. (B) The percentage of F4/80-positive cells was detected by flow cytometry.

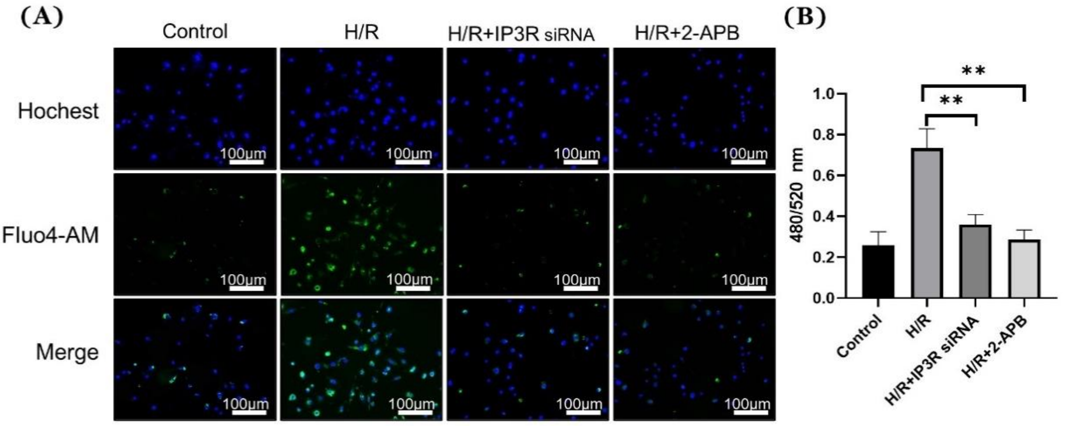

FIGURE S3 The increased calcium in H/R-induced liver macrophages is mainly released from ER KCs that were treated with H/R and blocked by IP3 siRNA (20 μM) or 2-APB (100 μM, 24 h). (A) The concentration of calcium was measured by immunofluorescence, where intracellular calcium was labeled with Fluo-4 AM (green) and the nucleus was labeled with Hoechst stain (scale bar, 100 μm). (B) The relative concentration of calcium in each group was measured with a fluorescence microplate reader. All data are shown as the mean ± SD (n=6). ***P< 0.001, **P < 0.01 and *P< 0.05.
